# Interplay between the Lung Microbiome, Pulmonary Immunity and Viral Reservoirs in People Living with HIV under Antiretroviral Therapy

**DOI:** 10.3390/v14112395

**Published:** 2022-10-29

**Authors:** Zihui Wang, Mohammad-Ali Jenabian, Yulia Alexandrova, Amélie Pagliuzza, Ron Olivenstein, Suzanne Samarani, Nicolas Chomont, Steven W. Kembel, Cecilia T. Costiniuk

**Affiliations:** 1 Département des Sciences Biologiques, Université du Québec à Montréal (UQAM), Montreal, QC H2X 1Y4, Canada; 2 Département de Microbiologie, Infectiologie et Immunologie, Université de Montréal, Montreal, QC H3T 1J4, Canada; 3 Infectious Diseases and Immunity in Global Health Program, Research Institute of MUHC, Montreal, QC H4A 3J1, Canada; 4 Department of Microbiology and Immunology, McGill University, Montreal, QC H3A 2B4, Canada; 5 Centre de Recherche du CHUM, Montreal, QC H2X 0A9, Canada; 6 Division of Respirology, MUHC, Montreal, QC H4A 3J1, Canada; 7 Division of Infectious Diseases and Chronic Viral Illness Service, McGill University Health Centre, Montreal, QC H4A 3J1, Canada

**Keywords:** HIV, pulmonary immunity, microbiome, lungs, HIV reservoirs

## Abstract

Pulmonary dysbiosis may predispose people living with HIV (PLWH) to chronic lung disease. Herein, we assessed whether intrapulmonary HIV reservoir size and immune disruption are associated with reduced bacterial lung diversity in PLWH. Bacterial DNA was extracted and PCR-amplified from cell-free bronchoalveolar lavage (BAL) fluid from 28 PLWH and 9 HIV-negative controls. Amplicon sequence variant (ASV) relative abundances and taxonomic identities were analyzed using joint species distribution modeling. HIV-DNA was quantified from blood and pulmonary CD4+ T-cells using ultra-sensitive qPCR. Immunophenotyping of BAL T-cells was performed using flow cytometry. Lung microbiome diversity was lower in smokers than non-smokers and microbiome composition was more variable in PLWH than HIV-negative individuals. Frequencies of effector memory BAL CD4+ and CD8+ T-cells positively correlated with abundance of several bacterial families while frequencies of BAL activated CD4+ T-cells negatively correlated with abundance of most lung bacterial families. Higher HIV-DNA levels in blood, but not in BAL, as well as frequencies of senescent CD4+ T-cells were associated with reduced bacterial diversity. These findings suggest that HIV infection may weaken the relationship between the lung microbiome and smoking status. Viral reservoir and immune activation levels may impact the lung microbiome, predisposing PLWH to pulmonary comorbidities.

## 1. Introduction

Due to antiretroviral therapy (ART), lifespans of people living with HIV (PLWH) now approach those of uninfected individuals [1] and there has been a shift in the spectrum of pulmonary diseases observed. While PLWH remain at higher risk for bacterial pneumonias, respiratory viruses and Tuberculosis than uninfected individuals, they also have a higher burden of non-infectious lung disorders, including chronic obstructive pulmonary disease (COPD) and asthma [2,3,4]. Furthermore, the rate of lung cancers exceeds that in HIV uninfected persons [5,6]. Furthermore, we recently demonstrated greater HIV reservoir size (HIV-DNA) within the lung mucosa compared to blood despite a decade of long-term suppressive ART [7,8].

The microbiome consists of a spectrum of microorganisms including bacteria, viruses and fungi which are intimately interconnected with each other and with the host [9]. Its composition is influenced by body site, diet, antibiotics, lifestyle, socioeconomic status, pollution and environmental factors [9]. Dysbiosis is an imbalance or maladaptation of bacterial communities and is associated development and progression of lung disease [10]. In PLWH, the lung microbiome dysbiosis increases risk of adverse outcomes, including pneumonia and respiratory infections, chronic lung diseases such as asthma and COPD, pulmonary fibrosis and lung cancer [11,12,13,14,15]. However, there is a gap in our understanding of the role of lung dysbiosis in pulmonary disease in PLWH. While it is accepted that the diversity and composition of the lung microbiome differs in PLWH versus uninfected people, the interplay between the lung microbiome, pulmonary HIV reservoir size and lung immunity has not been previously explored. Furthermore, the impact of smoking on these parameters is unclear. In the present study, we assessed whether intrapulmonary HIV reservoir size and immune disruption are associated with a reduction in bacterial lung diversity in PLWH, which may predispose PLWH to chronic lung disease.

## 2. Materials and Methods

### 2.1. Study Population

Bronchoalveolar lavage (BAL) fluid was obtained from 28 individuals (14 smokers, 14 non-smokers) with well-controlled HIV on suppressive ART (undetectable viral load for ≥3 years) and 9 HIV-negative controls (4 smokers, 5 non-smokers) as described in Table 1. Tobacco smokers endorsed daily tobacco smoking for at least 3 years. Non-smokers denied any smoking of any substances. All individuals were enrolled at McGill University Health Centre (Montreal, Canada). All participants recruited did not exhibit any respiratory symptoms or active infection.

### 2.2. Ethical Consideration

This study was ethically approved by the Research Institute of the McGill University Health Centre (#15-031), Université du Québec à Montréal (#602) and CHUM-Research Centre (#15-180). All participants signed a written informed consent.

### 2.3. Bronchoalveolar Lavage and Blood Collection

Bronchoscopies were performed to obtain up to 100 mL of BAL fluid. BAL and PBMCs underwent separation of the cellular vs. cell-free component as we previously published [7,8,16].

### 2.4. Flow Cytometry Phenotyping of CD4 and CD8 T-cell Subsets

Frequency of different CD4+ and CD8+ T cell subsets were defined via multi-parameter flow cytometry (BD Fortessa X-20) in total BAL cells or PBMCs as we previously described [7,16]. To eliminate dead cells from the analysis, we stained cells with Aqua viability stain (Invitrogen, Waltham, MA, USA). Expressions of CD8+, CD4+ and/or CD8+, CD45RA+, CD28+ were used to enumerate naïve (CD45RA^+^ CD28^+^), central memory (CD45RA^-^ CD28^+^), effector memory (CD45RA^-^ CD28^-^) and terminally differentiated (CD45RA^+^ CD28^-^) cells. Levels of CD8+ and CD4+ T cell immune activation (CD38+ HLADR+), and senescence (CD28- CD57+) were assessed on CD4+ and CD8+ T cell subsets.

### 2.5. HIV-DNA Quantification

Total DNA from PBMCs and total BAL cells was extracted using the Qiamp DNA mini kit (Qiagen) according to manufacturer’s instructions [7,8,16] HIV DNA quantification (LTR-gag) was performed in triplicate wells using an ultra-sensitive PCR adapted protocol as we previously described [7,8]. Only samples for which at least 3000 cells were available were used in the analysis.

### 2.6. Sequence Data Collection and Analysis

#### 2.6.1. Sequencing

Cell-free BAL specimens were stored at −80 °C until analyzed in batch. Microbial DNA was first extracted using the QIAGEN DNeasy PowerMax Soil kit following the manufacturer’s instructions. Then, PCR amplification was performed targeting the bacterial 16S rRNA gene V5-V6 region using primers 799F and 1115R [17]. We conducted PCR in a triplicate 25 μL mixture containing 5 μL 5× Phusion HF Buffer (Thermo Fisher Scientific, Frederick, MD, USA), 0.5 μL dNTPS (10 μmol/L each), 0.75 μL DMSO, 0.5 μL each primer (10 μmol/L), 0.25 μL Phusion Hot Start II polymerase (2 U/μL) (Thermo Fisher Scientific), 1 μL DNA template and 16.5 μL molecular-grade H_2_O. PCR reactions were performed using the following condition: 30 s initial denaturation at 98 °C, followed by 35 cycles of 15 s at 98 °C, 30 s at 64 °C, and 30 s at 72 °C, with a final 10 min elongation at 72 °C. PCR products were first normalized using a SequalPrep Normalization kit (Thermo Fisher Scientific), then pooled and purified using AMPure (Beckman Coulter Life Sciences, Brea, CA, USA) to avoid contaminants. After that, we prepared the DNA library by mixing equimolar concentrations of DNA for each sample and sequenced the DNA using Illumina MiSeq reagent kit v3 (Illumina, Hayward, CA, USA) [8].

#### 2.6.2. Bioinformatics

We used DADA2 (version 1.20) to process sequences, identify amplicon sequence variants (ASVs) and assign ASVs to taxonomic taxa [18]. We trimmed sequences to remove primers and low-quality nucleotide, keeping nucleotides at positions 17–283 bp and 21–179 bp for forward and reverse DNA sequences, respectively. Then, we inferred sequence error rates and inferred ASVs (amplicon sequence variants with error-corrected identical sequences) using default parameter values of DADA2 [18] with the following exceptions: The paired reads were merged with a minimum overlap of 15 nucleotides. Non-target sequences and chimeras were removed and ASV sequences were annotated taxonomically by comparison with the SILVA SSU r138 database [19]. A total of 1,377,448 sequences of 938 ASVs were obtained from 37 samples. The number of sequences per sample varied among samples, ranging from 2881 to 102,776 sequences/sample. Sample DNA quantities were normalized prior to sequencing, so this variation in sequence depth per sample is not necessarily biologically meaningful. To eliminate the bias induced by varying sequencing depth, we randomly rarefied the ASV matrix to 2881 sequences per sample using vegan R package [20], leading to the removal of 83 extremely rare ASVs after rarefaction. The rarefaction threshold was sufficient to capture most ASV diversity in our samples, as evidenced by a rarefaction curve which indicated the number of ASVs per sample reached a plateau at approximately 3000 sequences per sample (Figure S1). Rarefied ASV composition data was used for all subsequent analysis.

#### 2.6.3. Statistics

For immunological data, GraphPad prism V 6.01 (San Diego, CA, USA) was used to perform statistical analyses. The Wilcoxon matched-pair signed rank test and Mann–Whitney test were used to compare paired and unpaired variables, respectively. In the text, reported results follow the mean +/− standard error format.

For microbiome data, we first calculated the Shannon diversity index for each lung bacterial community. The Shannon index considers the number of ASVs (richness) and their relative abundance (evenness) where a lager value of the index indicates a more diverse community with more ASVs and/or a more equitable distribution of abundance among ASVs. We quantified links between Shannon diversity and variables including smoking and HIV status, CD4+/CD8+ count and ratio in PBMC, cellular immune activation markers, HIV reservoir size and immunosenescence separately using univariate linear regressions.

To quantify the variation in community composition of lung bacteria in relation to smoking and HIV status, we applied non-metric multidimensional scaling (NMDS) ordination of the Bray–Curtis dissimilarity among lung bacteria samples. The NMDS ordination assigns coordinates to each sample based on the dissimilarity among samples, and the distance between samples in the ordination space indicate the dissimilarity of their lung bacteria communities. Ellipses were added to indicates 95% confidence intervals around the clusters of bacterial communities for four study groups. Permutational multivariate analysis of variance (PERMANOVA) was used to test the effect of smoking and HIV status on lung bacterial community composition. We then measured the multivariate dispersion (variance) in community composition within these groups by calculating the average distance of samples to the group centroid [21]. The ‘distance-to-centroid’ index indicates the variability of community composition within the group, with larger values indicating that community composition varies widely among samples within the group. We tested if the distance-to-centroid differed among the four groups using Tukey’s test implemented in the vegan R package [20]. We also modeled the distance-to-centroid index as function of HIV and smoking status in a linear regression to test if PLWH and smokers have higher variability in lung bacteria community composition than non-smoker HIV-negative individuals.

To explore if the physiological variables (CD4+/CD8+ count and ratio, cellular immune activation markers, HIV reservoir size and immunosenescence) had a potential influence on lung bacterial community composition, we conducted distance-based redundancy analysis (dbRDA) which performs an ordination to measure the variation in Hellinger-transformed bacterial community composition explained by different variables. The dbRDA ordination of each sample was plotted using the ggord R package where the direction and strength of constraints influencing bacterial communities were depicted as arrows and the significance of constraints was tested using an ANOVA-like permutation test.

We conducted joint species distribution modeling (JSDM) to explicitly test the influence of immune activation variables on lung bacterial communities, in particular on the abundance of major bacterial families. We excluded rare bacterial families that were present in fewer than 5 samples as the estimation of model parameters for these rare families in the JSDM framework is challenging. We used the abundance of each bacterial family as the response variables in these models. Due to the nature of sequence count data, we used Poisson models with non-fixed dispersion parameters to model abundances. For the explanatory variables, we included a limited number of variables in the model to avoid overfitting. We included smoking as a predictor in all JSDMs due to the important association of this variable on the abundance of most bacterial families. We tested different combination of immune variables and selected the variables that had significant impacts on the abundance of most bacterial families, which included smoking, the frequencies of effector memory BAL CD4+ T-cells, the frequencies of BAL CD4+ T-cells expressing HLA-DR+ and CD38+ HLA-DR+. Model fitting was conducted using the R-package Hmsc assuming the default prior distributions [22]. We sampled the posterior distribution with four Markov Chain Monte Carlo (MCMC) chains, each of which was run for 37,500 iterations, of which the first 12,500 were removed as burn-in. The chains were thinned by 100 to yield 250 posterior samples per chain. We examined MCMC convergence by calculating the potential scale reduction factors of the model parameters. Our models showed a satisfactory MCMC convergence (the potential scale reduction factors for the β-parameters that measure the responses of the bacterial families to immune variables were on average 1.02 (maximum 1.1)). After that, we calculated the effect coefficients of explanatory variables on bacteria abundance for each bacterial family, including the intercept of the model which indicates the estimated abundance of each family when values of all explanatory variables were set equal to zero. We used linear scatter plots to show some examples of how bacteria abundance changed with explanatory variables though the abundance data does not fit normal regression and were modelled in the JSDM approach using a Poisson model.

## 3. Results

### 3.1. Participant Characteristics

37 participants were enrolled, including 28 HIV+ (14 smokers and 14 non-smokers) vs. 9 HIV- participants (4 smokers and 5 non-smokers), as shown in Table 1 and Supplementary Table S1. PLWH participants and uninfected controls did not significantly differ with respect to age, sex, ethnicity, or smoking status. For PLWH, the medium duration of HIV infection was 17 years (Interquartile range (IQR) 12.5, 24.8) and participants had suppressed viral loads for a median of 9 years (IQR 7, 10). Immunological clinical data and cellular immune activation markers are summarized in Table 1. As a limited number of measurements were obtained for some variables due to inter-individual variation in numbers of purified BAL cells and low T-cell frequencies, it was not possible to measure all physiological variables for all samples; only samples with data available for each variable were included in the analyses.

### 3.2. Sequence Data Summary

**Figure 1:**
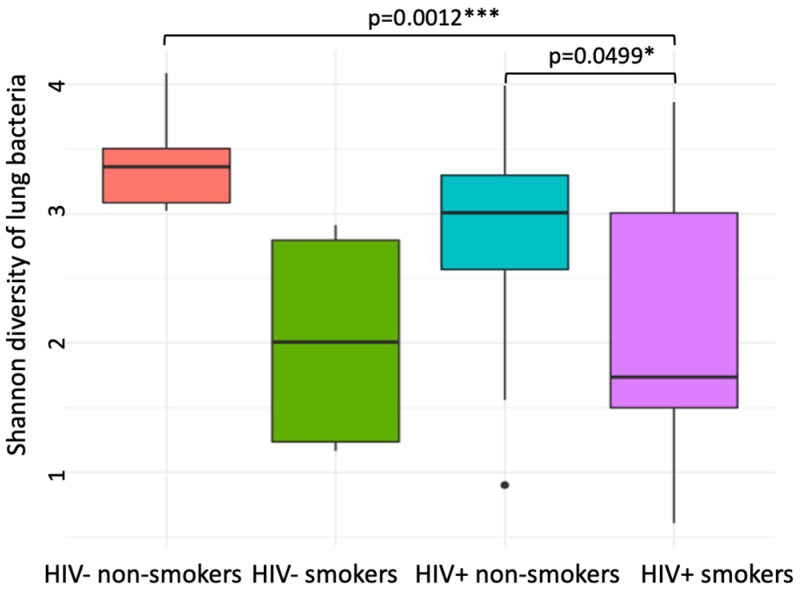
Lung bacterial community alpha diversity (Shannon index of relative abundance of amplicon sequence variants [ASVs] per sample) across HIV and smoking status. A two-way ANOVA was performed to test the impact of smoking and HIV status on bacteria diversity, showing significant effect of smoking (*p* = 0.005) but no effect of HIV status and its interaction with smoking (*p* = 0.32 and *p* = 0.4, respectively). Pairwise *t*-tests were performed to test for differences among the four groups and the significant differences are shown (* *p* < 0.05; *** *p* < 0.001).

A total of 106,570 high-quality read sequences from 855 ASVs were obtained from the 37 samples. Most of these ASVs were annotated taxonomically to at least the bacterial genus level; 94% of ASVs were annotated as belonging to 76 bacterial families and 86% to 122 bacteria genera, while only 17% of ASVs were identified to the species level. *Bacteroidota* were the dominant phylum, accounting for 30% of total sequence abundance. *Prevotellaceae* and *Prevotella* were the most abundant family and genus, respectively, accounting for 26% and 15% of total sequence abundance (Figure S2). The number of ASVs varied widely among samples ranging from 5 to 125 ASVs per sample, with a marginally significant reduction of ASV richness in smokers compared with non-smokers (mean ± SEM: 55 ± 5 vs. 34 ± 5 ASVs/sample for smokers and non-smokers, respectively; ANOVA, *p* = 0.07) but no difference in richness between HIV- and HIV+ (46 ± 5 vs. 44 ± 5; ANOVA, *p* = 0.86). Smoking significantly reduced the Shannon diversity index of lung bacterial communities (ANOVA, *p* = 0.005), while HIV status had no impact on the Shannon index (ANOVA, *p* = 0.32, Figure 1).

### 3.3. Compositional Analysis of Pulmonary Microbiota

**Figure 2:**
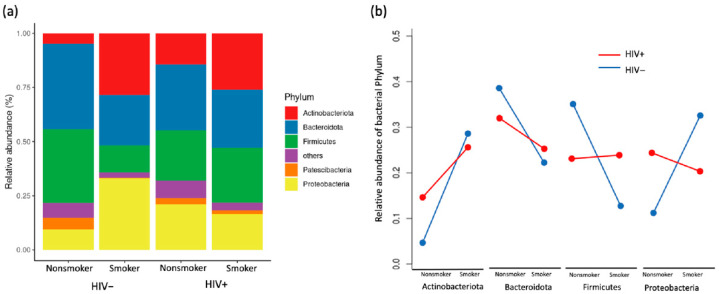
The composition of lung bacterial phyla (**a**) and the response of major phyla to smoking (**b**) for PLWH and Non-HIV samples.

**Figure 3:**
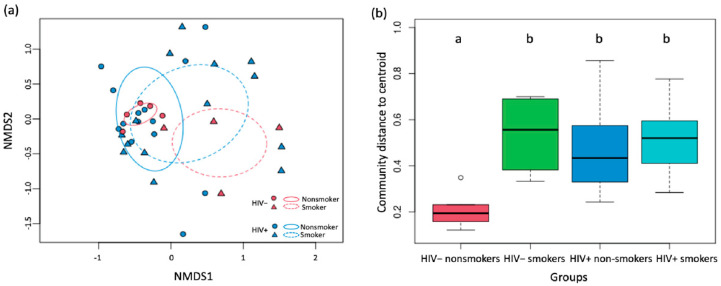
Non-metric multidimensional scaling (**a**) and within-group variation of lung bacterial community (**b**) across HIV and smoking status. In (**a**), samples from HIV- and HIV+ were colored in blue and red, respectively, and symbols for smokers and nonsmokers were circles and triangles, respectively. Ellipses (95% confidence intervals) were drawn around samples from the four groups. PERMANOVA showed significant differences in microbiome composition between smokers and non-smokers for HIV- (red, *p* = 0.13) but not for PLWH (blue, *p* = 0.44). In (**b**), distance-to-centroid index was calculated for samples in each group and the different letter labels (‘a’ vs. ‘b’) indicate a significant difference while same letters indicate no significant difference between groups at *p* < 0.05 by Tukey’s test.

Smoking altered the composition of bacterial phyla by reducing the abundance of *Bacteroidetes* and *Fusobacteria* and promoting that of *Actinobacteriota* and *Proteobacteria* for uninfected individuals, while for PLWH, the bacterial phyla composition was similar between smokers and nonsmokers (Figure 2a). We observed a large difference in phyla abundance between smokers and non-smokers in HIV- but not in HIV+, suggesting that lung bacteria phyla were less affected by smoking in PLWH than uninfected individuals (Figure 2b). Using non-metric multidimensional scaling (NMDS) to visualize the variation in community composition of bacterial families among samples, we show that bacterial community composition differed between smokers and non-smokers for HIV- (PERMANOVA, *p* = 0.013) but this was not observed in PLWH (*p* = 0.44, Figure 3a), suggesting that smoking induced changes in lung microbiomes but this change was diminished by HIV infection. Multivariate dispersion analysis of lung bacterial communities showed different degrees of within-group variation across HIV and smoking status. Nonsmoker uninfected individuals showed the smallest within-group variation in community composition, suggesting that their lung bacterial communities are relatively homogenous among individuals and stable (Figure 3b). Both smoking and HIV+ status altered lung bacterial community composition and increased the within-group compositional variability (Linear regression, *p* = 0.006 and *p* = 0.008 for smoking and HIV, respectively). For example, PLWH have higher compositional variability of lung microbiome than HIV- regardless of whether they were smokers or not, indicating that HIV infection creates heterogeneous communities that differ greatly among infected individuals (Figure 3b). The within-group variation for HIV+ individuals was so large that it diminished the among-group difference between HIV+ and HIV- groups and made HIV status insignificant in affecting bacterial composition (PERMANOVA, *p* = 0.71).

### 3.4. Association between Lung Microbiota, Pulmonary Immune Activation and Immunosenescence

**Figure 4:**
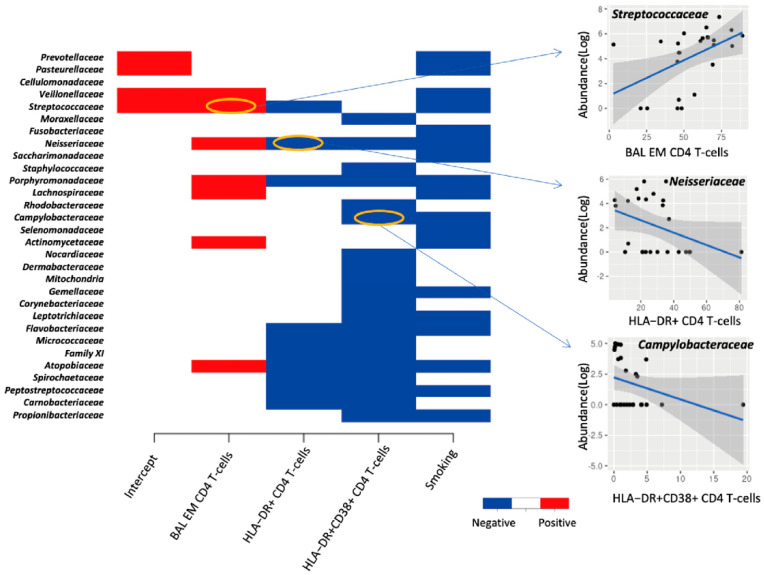
Effect of coefficients of the immune activation markers on the abundance of lung bacterial families. Each row represents a bacterial family, and the column represents the immune activation variables. A significant correlation supported by at least 95% posterior probability was depicted in either blue or red. Specifically, we presented three bacterial families as examples to show the correlation between bacterial abundance and immune activation variables.

**Figure 5:**
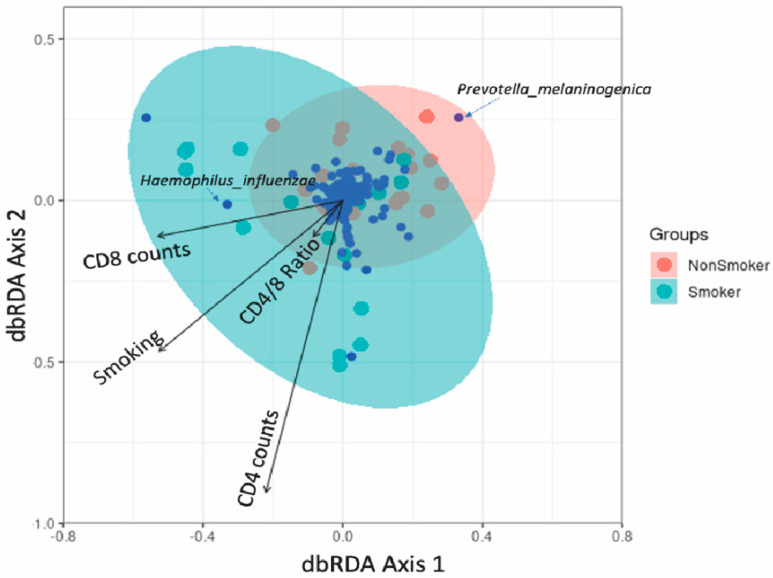
Distance-based redundancy analysis (dbRDA) on the lung bacterial community composition with explanatory variables of (CD4+ and CD8+ counts. Hellinger-transformed community data was ordinated as circles of either pink (nonsmoker) or cyan (smoker) and bacterial ASVs were presented as the small blue circles. The arrows represent the influence of constraining variables on bacterial communities where a small angle between two arrows indicated a similar influence of the two constraints on community composition. The relative position of arrows relative to the blue points (bacterial ASVs) suggests the impact of constraints on the abundance of ASVs and blue points near to the arrow line in the same/opposite direction indicates positive/negative impacts, e.g., *Prevotella melaninogenica* is located nearly opposite to the smoking arrow, suggesting that smokers have less *Prevotella melaninogenica* relative to non-smokers. 37 samples were included.

**Figure 6:**
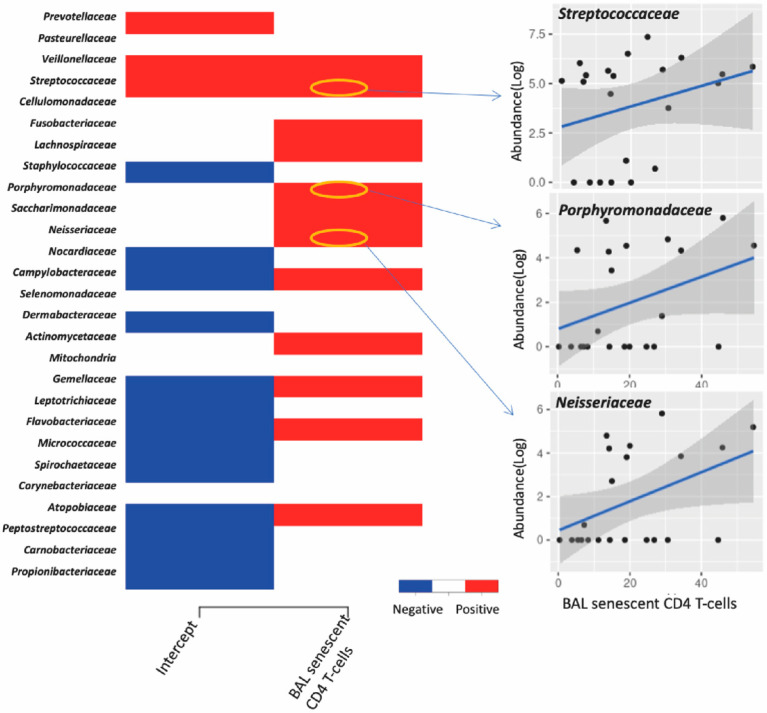
Effect coefficients of senescent CD4+ T-cells on the bacterial families. Three bacterial families were presented as examples whose abundance was influenced by senescent CD4+ T-cells (sample size = 22).

**Figure 7:**
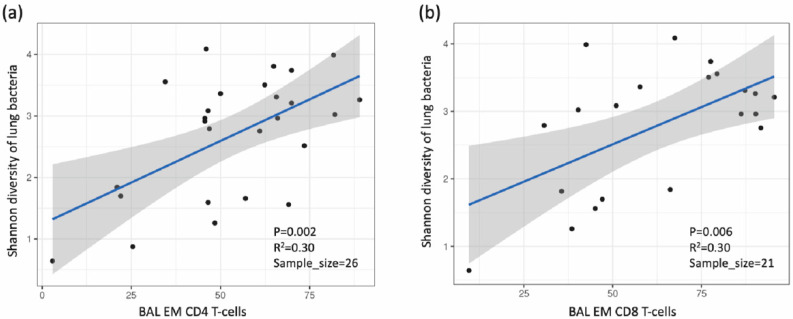
Relationship between microbial diversity vs. (**a**) CD4+ and (**b**) CD8+ BAL EM T-cells.

In PLWH, the premature onset of age-related illnesses is often attributed to immune activation and senescence, in a process known as “inflammageing” [23,24,25]. Joint species distribution models show that smoking significantly reduced the abundance of most bacterial families compared with their abundance in non-smokers. Additionally, increased frequencies of BAL activated CD4+ T-cells expressing HLA-DR+ and CD38+ HLA-DR+ were linked with decreased abundance of most bacterial families, and the frequencies of effector memory BAL CD4+ T-cells was positively correlated with the abundance of several bacterial families in the lungs (Figure 4). However, neither immune activation nor immune senescence markers of CD8+ T-cells were associated with the abundance of lung bacterial families (data not shown). We also found that the CD8+ cell counts and CD4+/CD8+ ratio in PBMC influence the abundance of a few but very abundant lung bacterial families such as *Prevotellaceae*, *Veillonellaceae* and *Streptococcaceae* (Figure S3). The effect of increased CD4+/CD8+ ratio on these dominant bacterial families altered the lung community towards a composition that resembled that found in smokers, characterized by less abundant *Prevotella melaninogenica* and more abundant *Haemophilus influenzae* (Figure 5). Greater proportions of senescent CD4+ T-cells were associated with higher lung bacterial diversity. Increased numbers of senescent CD4+ T-cells were associated with a reduction in the abundance of many bacterial families such as *Streptococcaceae, Porphyromonadaceae* and *Neisseriaceae* (Figure 6). With regard to CD4+ and CD8+ T-cell subsets, there was a positive relationship between Shannon microbial diversity and frequencies of both CD4+ and CD8+ BAL effector memory T-cells (Figure 7).

### 3.5. Association between Lung Microbiota and HIV Reservoir Size within the Lungs

**Figure 8:**
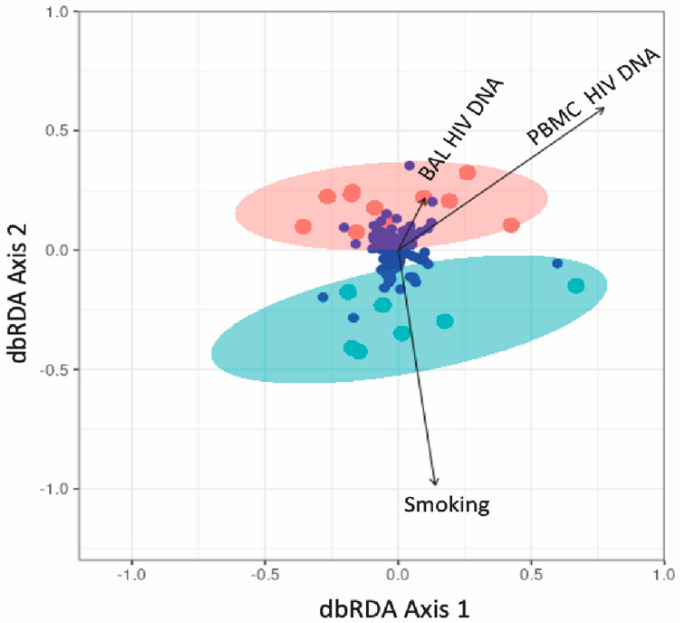
Distance-based redundancy analysis (dbRDA) on the lung bacterial community composition with explanatory variables of smoking and HIV-DNA levels (sample size = 18). Hellinger-transformed community data was ordinated as circles of either pink (nonsmoker) or cyan (smoker) and bacterial ASVs were presented as the small blue circles. The arrows represent the influence of constraining variables on bacterial communities where a small angle between two arrows indicated a similar influence of the two constraints on community composition.

**Figure 9:**
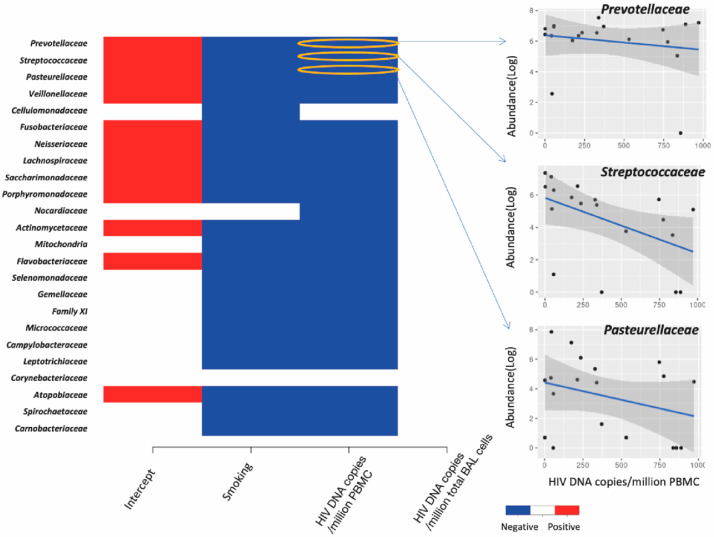
Effect of coefficients of HIV-DNA levels in PBMC and BAL on the abundance of lung bacterial families. Each row represents a bacterial family, and the column represents the HIV-DNA levels. A significant correlation supported by at least 95% posterior probability was depicted in either blue or red. Specifically, we presented three bacterial families as examples to show the correlation between bacterial abundance and HIV-DNA levels. 18 samples were included.

We found a negative relationship between lung bacterial diversity and HIV-DNA levels in PBMC (Linear regression, estimate = −0.002, *p* = 0.029), while no relationship was detected between bacterial diversity and HIV-DNA in BAL (*p* = 0.61) (data not shown). The HIV-DNA levels in PBMC also had a significant impact on the community composition of lung bacteria (ANOVA test on distance-based redundancy analysis, F = 1.22, *p* = 0.02, Figure 8) while that in BAL did not (F = 0.8, *p* = 0.89). Moreover, higher levels of HIV-DNA in blood but not in BAL decreased the abundance of most lung bacteria including *Prevotellaceae*, *Streptococcaceae* and *Pasteurellaceae* (Figure 9).

## 4. Discussion

Pulmonary dysbiosis is associated with various respiratory diseases [26], and PLWH suffer from a high burden of chronic lung disease despite ART [27,28]. We found that smoking status altered the composition of BAL bacterial phyla compared to that of non-smokers, although in PLWH the bacterial phyla were similar between smokers and non-smokers. In people without HIV infection or lung disease, the bacterial phyla *Actinobacteria* and *Proteobacteria* were more abundant in smokers, while *Bacteroidetes*, *Fusobacteria* and *Patescibacteria* were less abundant in smokers [29,30,31]. Similarly, we also found that smoking was associated with reduced abundance of *Bacteroidetes* and *Fusobacteria* and increased abundance of *Actinobacteriota* in HIV uninfected persons. Although these results are predominantly descriptive at this stage, these findings nonetheless indicate an interesting relationship between lung microbiomes, pulmonary immunity and viral reservoirs in people living with HIV under antiretroviral therapy.

Using multivariate dispersion analysis of lung bacterial communities, we observed different amounts of within-group variation across HIV and smoking status. Both smoking and HIV+ status altered lung bacterial community composition and increased the within-group compositional variability, indicating that HIV infection and smoking both create heterogeneous communities that differ greatly among individuals. As anticipated, uninfected non-smokers showed the smallest within-group variation in composition, suggesting their lung bacterial communities are relatively homogenous and stable among individuals, compared with the more variable community composition among smokers and PLWH. It is possible that variation in microbiota induced by HIV status overshadows that induced by smoking. Alternatively, both smoking and HIV status may lead to decreases in bacterial diversity and shifts in community composition compared with uninfected non-smokers. This finding makes intuitive sense, since HIV infection and taking ART induce various effects on an individual’s immunological and physiological status. Similarly, tobacco smoke contains thousands of compounds which may also influence the intrapulmonary immune milieu [32], impacting the types of microorganisms that can thrive. However, findings on differences in the lung microbiota amongst smokers and PLWH vary across studies. Morris et al. reported that smoking did not significantly affect the lung microbiome of PLWH compared to healthy smokers vs. healthy non-smokers [33]. Meanwhile, Xu et al. found that PLWH had increased *Proteobacteria* levels and decreased *Bacteroides* and *Firmicutes* levels in BAL compared with HIV negative controls [34].

In both the lung and the gut, reduced microbial diversity is generally associated with disease for many diseases including COPD, atopic disease, colitis, and cystic fibrosis [35,36,37,38]. In studies on the gut microbiome, HIV infection is usually associated with reduced microbial alpha diversity [39]. In the lung microbiome, Twigg et al. found individuals with advanced HIV had reduced alpha diversity (richness and evenness) and greater beta diversity in BAL of the HIV-negative population [40]. Xu et al. found decreased microbial diversity in the HIV small airway epithelium versus HIV-uninfected persons [34]. Our results differed from these findings, as we found no effect of HIV infection but an effect of smoking on lung microbial diversity. However, for PLWH in our study, we found that lung microbiome diversity was lower in smokers than non-smokers, suggesting that HIV infection mediates the effect of smoking on the lung microbiota.

We observed a negative association between lung bacterial diversity and HIV-DNA levels in PBMC and found that HIV-DNA levels were associated with changes in the composition of lung bacterial communities. The relationship between HIV DNA and cellular activation has been consistently demonstrated [41], and thus the HIV reservoir may play a role in the development of chronic lung pathology by driving inflammation, with related impacts on the diversity and composition of lung microbial communities. While HIV infection did not influence microbial diversity, the observation that microbial diversity and composition were influenced by peripheral HIV reservoir size, and more cellular activation, suggests that reduced bacterial diversity is associated with disease severity if not disease state per se. In the BCN02 clinical trial, a proof-of-concept study, an immunogen was combined with latency-reversing agent romidepsin in early-ART treated PLWH and the microbiome analyzed to determine gut microbiome patterns associated with HIV control [42]. The Bacteroidales/Clostridiales ratio, as well as host immune-activation signatures, inversely correlated with HIV-1 reservoir size, therefore making this ration a gut microbiome signature associated with HIV-1 reservoir size and immune-mediated viral control after ART interruption [42]. In another anatomical HIV reservoir, the male genital tract, the relationship of semen bacteria with HIV infection, semen cytokine levels, and semen viral load was examined. HIV infection was associated with decreased semen microbiome diversity and richness, which were restored after six months of ART [43]. In HIV-infected men, semen bacterial load correlated with seven pro-inflammatory semen cytokines and was associated with semen VL. Semen bacterial load was also directly linked to the semen HIV VL [43]. Thus, our findings agree with these studies in suggesting that the HIV reservoir appears to play a role in influencing microbiome diversity and composition.

Chronic immune activation is a hallmark of HIV infection and is thought to drive chronic systemic diseases [44]. Supporting this hypothesis, we found direct links between variables related to immune activation and lung microbiota; the Shannon diversity of lung bacteria was positively correlated with frequencies of effector memory BAL CD4+ T-cells. Effector memory cells quickly upregulate effector function and express homing receptors to travel to nonlymphoid sites of inflammation [45]. In addition, they express high levels of the gut homing molecule α_4_β_7_ integrin and chemokine receptors that target these cells to nonlymphoid tissues [45]. While all memory CD4+ T cell subsets harbor replication-competent HIV, effector memory T cells harbor more intact HIV-1 provirus than either central memory or terminally differentiated T cells [46]. Moreover, effector memory cells contain the highest level of inducible HIV, supporting their role in HIV persistence [47]. Therefore, when effector memory CD4+ and CD8+ T-cells migrate into lungs, such as during smoking, production of pro-inflammatory cytokines may promote tissue inflammation and damage. Although we did not find an association between frequencies of effector memory cells and reservoir size, it is possible that a relationship may have been observed with a larger sample size.

Our team previously demonstrated that levels of HLA-DR+CD38+ BAL CD4+ T-cells remain higher in ART-treated PLWH compared to healthy controls [7]. In the current study, increased frequencies of BAL activated CD4+ T-cells expressing HLA-DR+ and CD38+HLA-DR+ were linked with decreased abundance of most bacterial families, and the frequencies of effector memory BAL CD4+ T-cells was positively correlated with the abundance of several bacterial families in the lungs. HLA-DR+ and CD38+HLA-DR+ are markers of cellular activation and increased peripheral cellular activation is associated with greater CD4+ T-cell loss and progression to HIV and blood viral load [23]. We also found that the CD8+ cell counts and CD4+/CD8+ ratio in PBMC influence the abundance of a few but very abundant lung bacterial families such as *Prevotellaceae*, *Veillonellaceae* and *Streptococcaceae*. The effect of increased CD4+/CD8+ ratio on these dominant bacterial families altered the lung community towards a composition that resembled that found in smokers, characterized by less abundant *Prevotella melaninogenica* and more abundant *Haemophilus influenzae*. A low peripheral blood CD4+/CD8+ ratio is a poor prognostic factor and has been associated with higher cell-associated HIV DNA level in PLWH on ART [48].

We found that a greater proportion of senescent CD4+ T-cells were associated with higher lung bacterial diversity. Increased numbers of senescent CD4+ T-cells were also associated with a reduction in the abundance of many bacterial families such as *Streptococcaceae, Porphyromonadaceae* and *Neisseriaceae*. CD4+ T-cells play a critical role in achieving a regulated effective immune response to pathogens. Meanwhile, senescent cells promote physiological dysfunction via their progressively changing into a proinflammatory profile and impaired immune response [49]. Cellular senescence is believed to be an important driver of chronic lung diseases, such as COPD and idiopathic pulmonary fibrosis [50].

Limitations of our study include the cross-sectional nature and relatively small sample size for smoking sub-groups. We also did not examine viral or fungal microbiomes and used a reservoir measure (HIV DNA) that is known to overestimate the size of the replication-competent HIV reservoir [51]. Furthermore, although we performed bronchoscopies, there is undoubtedly mixing with oral flora to some degree. Other groups have showed that increased abundance of fungal species, such as *Pneumocystis* in the HIV lung, can cause airway inflammation and pulmonary function decline [4,52,53]. Importantly, due to the observational nature of most studies of the lung microbiome, it is unknown whether dysbiosis is a cause or consequence of disease. Similarly, it is unknown whether it dysbiosis coexists along with other factors implicated in driving pathogenesis. Whether any similar microbiota signatures, or host immune activation signatures, correlate with pulmonary HIV reservoir size remains to be explored. Ideally larger studies of a longitudinal nature would be conducted with individuals at different stages of HIV disease, both prior to and following at least a year of ART. It is plausible that ART itself may impact the lung microbiome. Emtricitabine and tenofovir result in mitochondrial dysfunction, increased oxidative stress and cellular senescence [54,55]. Although no data suggests ART is a risk factor for lung diseases [56], one study demonstrated that ART had a stronger adverse effect than HIV on the epigenetic responses of alveolar macrophages in response to *Mycobacterium tuberculosis* [56]. Our study did not enable us to unravel the differential contribution of HIV infection and ART, since all individuals with HIV were on ART. One could examine BAL fluid from HIV-negative individuals, some taking HIV pre-exposure prophylaxis versus those not on any HIV pre-exposure prophylaxis, to decipher the role of ART on lung microbiome. Similarly, one could examine BAL fluid of smokers with and without HIV, before and after smoking cessation, to better understand the relationships between HIV and smoking statuses. Finally, we reported on CD4+ and CD8+ lymphocytes and their subsets, although neutrophils [57,58] and alveolar macrophages [59] are known to play major roles in the context of pulmonary disease. Work is ongoing to examine the role of alveolar macrophages and pulmonary neutrophils in the context of the lung HIV reservoir burden the inflammatory landscape within the lungs of PLWH.

Despite these limitations, we report for the first time on a potential relationship between intrapulmonary HIV reservoir markers, pulmonary immune perturbations and relationship with lung dysbiosis in PLWH who are both tobacco smokers and non-smokers. Findings will inform the design of future larger studies, involving characterization of alveolar macrophages, lung neutrophils and inflammatory markers, and eventually the identification of a lung microbiome signature associated with development of chronic lung disease in PLWH. These findings will also inform future interventional studies aimed at modifying the lung microbiome, and ultimately improving the health, of PLWH.

## Figures and Tables

**Table 1:** Cellular immune activation markers and sequencing data.

Parameter	HIV+ (*n* = 28)		*p*-Value	HIV- (*n* = 9)		*p*-Value
Blood T Cell Markers and HIV-DNA	Smoker (*n* = 14)	Non-Smoker (*n* = 14)		Smoker (*n* = 4)	Non-Smoker (*n* = 5)	
CD4+ T-cell count (cells/mm^3^; mean ± SD)	635.0 ± 260.9	556.2 ± 196.6	*p* = 0.6	753.5 ± 284.9	409.4 ± 151.0	*p* = 0.06
CD4+/8+ T-cell ratio	0.81 ± 0.41	0.75 ± 0.36	*p* = 0.8	3.50 ± 1.23	2.14 ± 0.42	*p* = 0.2
Nadir CD4+ T-cells (mean ± SD)	185.8 ± 88.58	218.0 ± 109.3	*p* = 0.4	-	-	
%CD4+ HLADR+ CD38+	1.21 ± 0.88	1.60 ± 0.64	*p* = 0.2	0.95 ± 0.68	1.67 ± 2.67	*p* = 0.6
%CD4+ CD57+ CD28-	1.85 ± 2.06	10.21 ± 19.92	*p* = 0.6	0.62 ± 0.37	0.29 ± 0.34	*p* = 0.2
%CD8+ HLADR+ CD38+	0.92 ± 0.45	1.77 ± 2.12	*p* = 0.6	1.65 ± 0.78	2.41 ± 2.30	*p* > 0.9
%CD8+ CD57+ CD28-	32.93 ± 7.92	22.17 ± 15.93	*p* = 0.3	5.75 ± 1.50	15.92 ± 13.99	*p* = 0.4
PMBC HIV DNA	198.5 ± 295.0	534.5 ± 322.1	*p* = 0.02	-	-	
**BAL T cell markers and HIV-DNA**						
% CD4+ T-cells	47.33 ± 16.63	52.44 ± 12.25	*p* = 0.3	32.78 ± 22.59	63.30 ± 25.49	*p* = 0.1
% CD8+ T cells	39.86 ± 15.46	40.15 ± 12.30	*p* = 0.9	49.03 ± 21.60	20.44 ± 14.49	*p* = 0.06
%CD4+ HLADR+ CD38+	2.57 ± 2.22	4.07 ± 5.6	*p* = 0.6	1.42 ± 2.32	1.63 ± 2.02	*p* = 0.5
%CD4+ CD57+ CD28-	15.39 ± 10.02	28.01 ± 17.28	*p* = 0.1	12.81 ± 6.54	19.47 ± 17.28	*p* = 0.7
%CD8+ HLADR+ CD38+	2.46 ± 1.44	4.13 ± 3.71	*p* = 0.6	1.66 ± 1.77	11.68 ± 7.63	*p* = 0.1
%CD8+ CD57+ CD28-	17.77 ± 2.66	35.26 ± 16.67	*p* = 0.08	14.13 ± 10.14	15.90 ± 5.39	*p* = 0.5
BAL HIV DNA	1070 ± 1220	8061 ± 14119	*p* = 0.1	-	-	
**Microbial community data**						
Shannon index (mean ± SD)	2.04 ± 1.10	2.82 ± 0.88	*p* = 0.049	2.02 ± 0.93	3.41 ± 0.43	*p* = 0.052
Richness index (mean ± SD)	36.35 ± 38.04	52.07 ± 30.79	*p* = 0.24	27.75 ± 7.13	61.40 ± 36.65	*p* = 0.11

## Data Availability

Not applicable.

## Supplementary Materials

The following supporting information can be downloaded at: www.mdpi.com/article/10.3390/v14112395/s1. Table S1: Participant characteristics. Figure S1: The rarefication curve for each of the 37 samples. With increasing number of sampled sequences, the number of ASVs increases and reach a plateau of ASV richness at around 3000 sequences. Figure S2: The composition of bacterial (a) phyla and (b) families across all 37 samples. To facilitate presentation, we only show bacterial taxa with relative abundance above 0.01. Figure S3: Effect coefficients of the CD4/CD8 count and ratio in PBMC on the abundance of lung bacterial families. Each row represents a bacterial family, and the column represents the variables. A significant correlation supported by at least 95% posterior probability was depicted in either blue or red. Specifically, we presented two families as examples to show the correlation between bacterial abundance and immune activation variables. Sample size = 36.
