# Supplementary material for: Interplay between the Lung Microbiome, Pulmonary Immunity and Viral Reservoirs in People Living with HIV under Antiretroviral Therapy
Source: Viruses. 2022 Oct 29;14(11):2395. doi: 10.3390/v14112395 (PMC9693210; doi:10.3390/v14112395)

Supplementary Table S1: Participant characteristics

*All clinic patients undergo Tuberculin Skin Test (TST) testing at initial clinic visit as part of routine care

**Although patient prescribed raltegravir + emtricitabine/ tenofovir- disoproxil and advised to take all three medications, he has decided himself to only take emtricitabine/ tenofovir- disoproxil

PMHx: Past medical history; PCP: *Pneumocystis carinii* pneumonia; VL: viral load

ID	Age, years	Sex, M/F	Ethnicity	PMHx of PCP or Tuberculosis (active or latent)*	Duration HIV (years)	Tobacco	Cannabis	CD4 count/ mm^3	CD4/CD8 ratio	CD8 count/mL	Nadir CD4 count/mL	ART regimen at time of bronchoscopy	Undetectable VL (years)
**People living with HIV, smokers**													
9	40	M	Caucasian	No	16	Yes	Yes	720	0.6	1200	unknown	emtricitabine tenofovir elvitegravir cobicistat	11
11	53	F	Caucasian	No	30	Yes	No	797	0.5	1635	65	abacavir lamivudine raltegravir	3
12	51	F	Caucasian	No	25	Yes	No	578	0.7	868	43	emtricitabine tenofovir elvitegravir cobicistat	12

												darunavir	
15	70	M	Black-Caribbean	No	14	Yes	No	430	0.6	668	186	abacavir dolutegravir lamivudine	5
17	36	F	Hispanic	No	15	Yes	No	530	0.9	604	232	abacavir dolutegravir lamivudine	12
18	63	M	Black-Caribbean	No	12	Yes	No	916	0.8	1213	232	abacavir lamivudine efavirenz	10
19	58	M	Black-African	Yes (previous active TB)	10	Yes	No	545	1.1	490	236	abacavir dolutegravir lamivudine	10
22	28	M	Black-Caribbean	No	15	Yes	No	903	1.1	724	251	emtricitabine tenofovir cobiciastat elvitegravir	8
35	62	M	Caucasian	No	28	Yes	Yes	899	1.8	501	Unknown	abacavir dolutegravir lamivudine	9
38	57	M	Caucasian	No	32	Yes	Yes	396	0.4	933	Unknown	emtricitabine tenofovir efavirenz	10
39	58	M	Caucasian	No	20	Yes	No	269	0.7	362	Unknown	Elvitegravir Cobicistat	8

												Emtricitabine<br>Tenofovir	
40	54	F	Caucasian	No	24	Yes	Yes	360	0.4	828	268	Atazanavir<br>Raltegravir<br>Emtricitabine<br>Rilpivirine<br>Tenofovir	6
41	58	M	Caucasian	No	31	Yes	Yes	412	0.4	1138	79	Raltegravir<br>Etravirine<br>Norvir<br>Darunavir	11
44	58	M	Caucasian	No	22	Yes	Yes	1135	1.4	800	266	Darunavir<br>Ritonavir<br>Abacavir/lami<br>vudine	10
**People living with HIV, non-smokers**													
10	62	M	Caucasian	No	9	No	No	634	0.6	1155	323	Abacavir<br>dolutegravir<br>lamivudine<br>lopinavir<br>ritonavir	8
13	51	M	Caucasian	No	9	No	No	365	0.4	1022	154	abacavir<br>lamivudine<br>raltegravir	9

14	45	M	Caucasian	Latent TB infection treated with isoniazid	14	No	No	429	0.8	561	276	elvitegravir rilpivirine tenofovir	3
16	46	M	Caucasian	No	25	No	No	531	0.4	1202	140	Abacavir lamivudine darunavir norvir	16
20	51	M	Caucasian	No	28	No	No	350	0.7	447	300	emtricitabine tenofovir raltegravir	4
21	53	M	Caucasian	No	15	No	No	917	1.1	864	379	emtricitabine tenofovir**	3
23	52	F	Black-Africa	No	16	No	No	430	0.9	460	212	Abacavir dolutegravir lamivudine	14
24	52	M	Caucasian	PCP pneumonia 9 years earlier	9	No	No	375	0.7	537	43	emtricitabine tenofovir nevirapine	9
34	52	F	Black-African	No	19	No	No	621	1.8	888	275	emtricitabine tenofovir atazanavir ritonavir	7
36	67	M	Caucasian	No	23	No	No	602	0.7	880	202	Abacavir lamivudine raltegravir	10

37	58	M	Caucasian	No	22	No	No	537	0.5	1033	Unknown	emtricitabine tenofovir raltegravir	10
42	51	M	Caucasian	No	18	No	No	541	0.5	1180	Unknown	emtricitabine tenofovir elvitegravir cobicistat	8
43	52	M	Black African	No	10	No	No	448	0.7	622	26	emtricitabine tenofovir elvitegravir cobicistat	10
45	57	M	Black African	No	8	No	No	1007	0.7	1543	295	Ritonavir Darunavir Maraviroc abacavir	7
**HIV-negative smokers**													
27	58	M	Caucasian	No	N/A	Yes	No	603	2.1	289	N/A	N/A	N/A
30	63	M	Caucasian	No	N/A	Yes	No	675	3.5	193	N/A	N/A	N/A
32	48	M	Caucasian	No	N/A	Yes	No	561	5.1	110	N/A	N/A	N/A
33	55	M	Caucasian	No	N/A	Yes	Yes	1173	3.3	355	N/A	N/A	N/A
**HIV-negative non-smokers**													
25	27	M	Caucasian	No	N/A	No	No	302	2.4	127	N/A	N/A	N/A
26	65	M	Caucasian	No	N/A	No	No	287	2.2	128	N/A	N/A	N/A
28	60	M	Caucasian	No	N/A	No	No	612	2.4	256	N/A	N/A	N/A

29	61	M	Caucasian	No	N/A	No	No	315	1.4	223	N/A	N/A	N/A
31	69	M	Caucasian	No	N/A	No (former firefighter)	No	531	2.3	227	N/A	N/A	N/A

Figure S1: The rarefication curve for each of the 37 samples. With increasing number of sampled sequences, the number of ASVs increases and reach a plateau of ASV richness at around 3000 sequences.

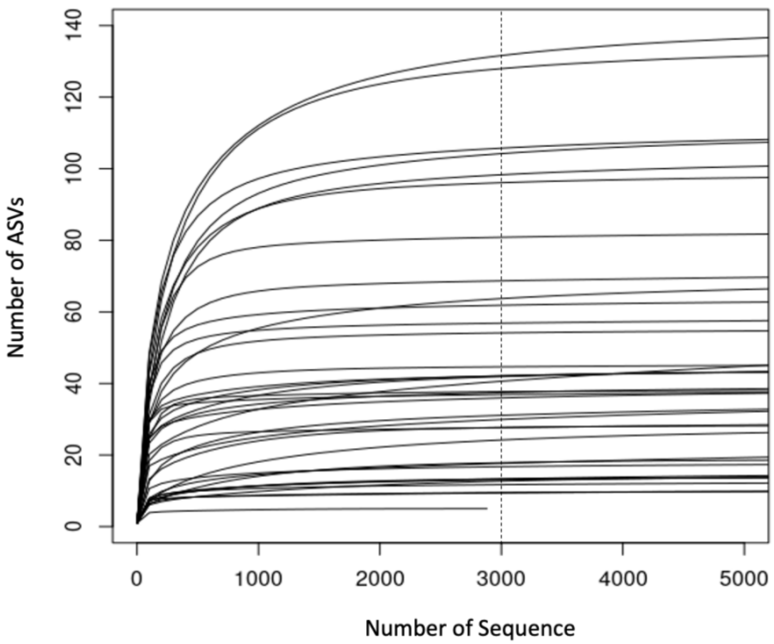

Figure S2 The composition of bacterial (a) phyla and (b) families across all 37 samples. To facilitate presentation, we only show bacterial taxa with relative abundance above 0.01

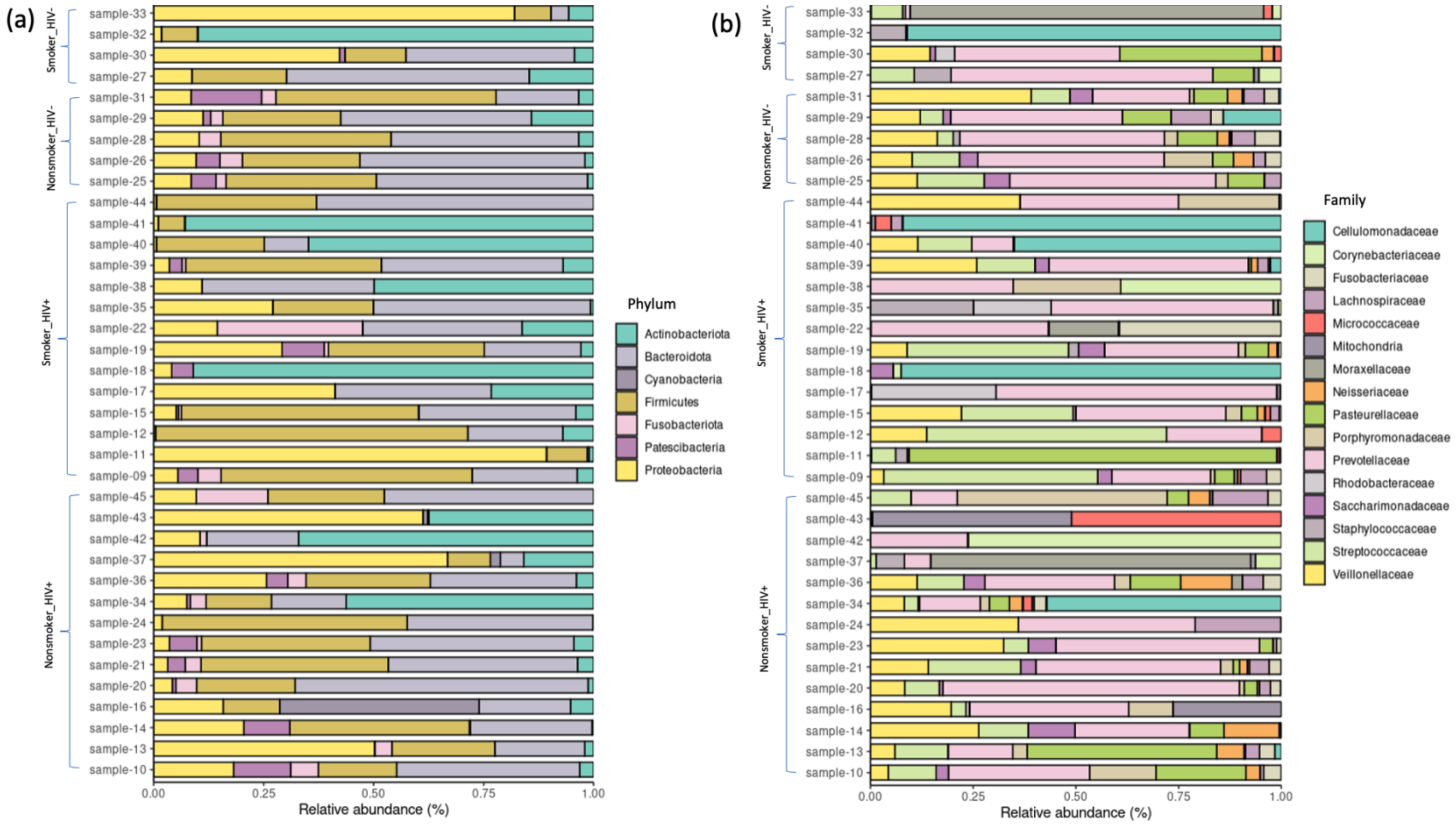

Figure S3 Effect coefficients of the CD4/CD8 count and ratio in PBMC on the abundance of lung bacterial families. Each row represents a bacterial family, and the column represents the variables. A significant correlation supported by at least 95% posterior probability was depicted in either blue or red. Specifically, we presented two families as examples to show the correlation between bacterial abundance and immune activation variables. Sample size = 36.

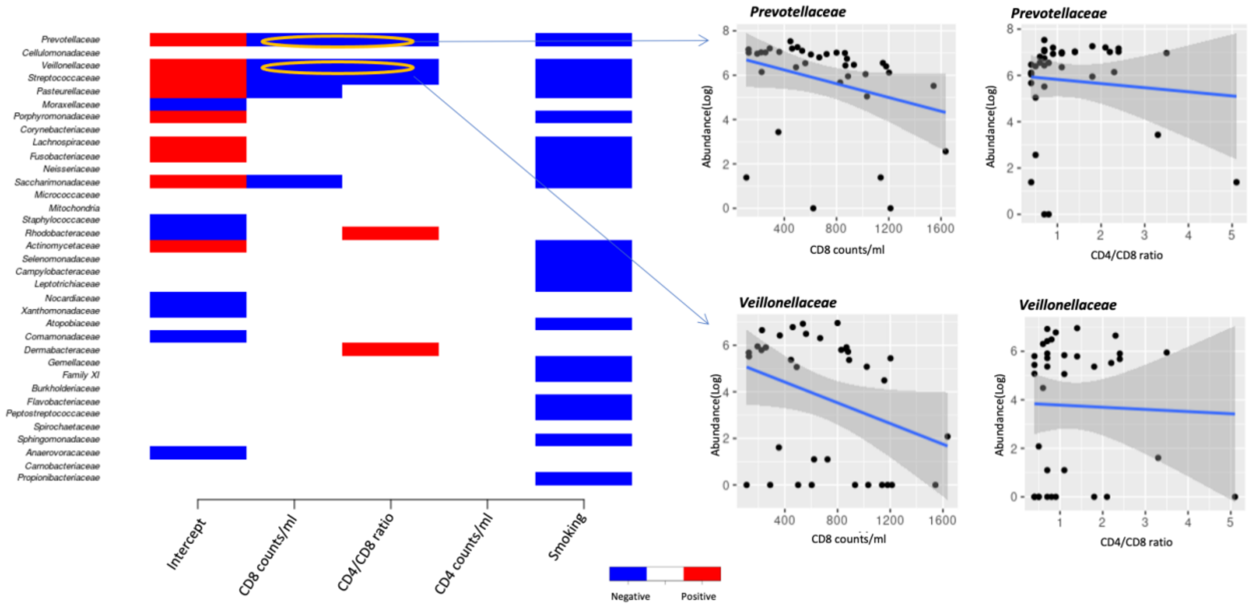

Supplement: Supplementary file 1 [file viruses-14-02395-s001.zip › viruses-1950275-supplementary.pdf]
